# Major Bleeding in Patients with Non-Valvular Atrial Fibrillation: Impact of Time in Therapeutic Range on Contemporary Bleeding Risk Scores

**DOI:** 10.1038/srep24376

**Published:** 2016-04-12

**Authors:** Marco Proietti, Keitaro Senoo, Deirdre A. Lane, Gregory Y. H. Lip

**Affiliations:** 1 University of Birmingham Institute of Cardiovascular Sciences, City Hospital, Birmingham, United Kingdom; 2 Department of Internal Medicine and Medical Specialties, Sapienza-University of Rome, Rome, Italy; 3 Aalborg Thrombosis Research Unit, Department of Clinical Medicine, Aalborg University, Aalborg, Denmark

## Abstract

Bleeding risk represents a major concern in anticoagulated patients with atrial fibrillation (AF). Several bleeding prediction scores have been described: HAS-BLED, ATRIA, HEMORR_2_HAGES and ORBIT. Of these, only HAS-BLED considers quality of anticoagulation control amongst vitamin K antagonist (VKA) users. We hypothesised that predictive value of bleeding risk scores other than HAS-BLED could be improved incorporating time in therapeutic range (TTR) in warfarin-treated patients. Of the 127 adjudicated major bleeding events, 21.3% of events occurred in ‘low-risk’ HAS-BLED category (1.8 per 100 patient-years), compared to higher proportions (≥50% of events; ~2.5 per 100 patient-years) in ‘low-risk’ categories for other scores. Only the ‘low-risk’ HAS-BLED category was associated with the absence of investigator-defined major bleeding events (OR: 1.46;95% CI: 1.00–2.15). ‘High’ or ‘medium/high’ risk categories for the HAS-BLED (p = 0.023) or ORBIT (p = 0.022) scores, respectively, conferred significant risk for adjudicated major bleeding events. On Cox regression analysis, adjudicated major bleeding was associated only with HAS-BLED (HR: 1.62;95% CI: 1.06–2.48) and ORBIT (HR: 1.83;95% CI: 1.08–3.09) ‘high-risk’ categories. Adding ‘labile INR’ (TTR < 65%) to ORBIT, ATRIA and HEMORR_2_HAGES significantly improved their reclassification and discriminatory performances. *In conclusion,* HAS-BLED categorised adjudicated major bleeding events in low-risk and high-risk patients appropriately, whilst ORBIT and ATRIA categorised most major bleeds into their ‘low-risk’ patient categories. Adding TTR to ORBIT, ATRIA and HEMORR_2_HAGES led to improved predictive performance for major bleeding.

Oral anticoagulant (OAC) therapy is the cornerstone of management in patients with atrial fibrillation (AF) for the prevention the risk of stroke and thromboembolism^1^,^2^,^3^. Bleeding risk represents the downside of treatment with anticoagulation, with a reported major bleeding incidence of 2.0 per 100 patient-years^4^. Therefore it is important to have simple and practical bleeding risk stratification tools for use in AF patients, to aid clinical decision-making^5^.

The HAS-BLED score^6^ is a simple, clinical risk factor based score which has been well validated in various cohorts^7^,^8^,^9^,^10^,^11^,^12^,^13^. Two other bleeding risk scores have been developed from large observational studies of AF populations and subsequently validated; the ATRIA^14^ and the HEMORR_2_HAGES^15^ scores. The HAS-BLED score has been shown to be as good as–or superior to–these other (and arguably more complicated) bleeding risk scores^11^,^16^,^17^,^18^. More recently, the ORBIT bleeding score was derived from a large contemporary AF prospective registry^19^,^20^, with the aim to propose a simpler score to be used for the assessment of bleeding risk in AF patients, irrespective of the type of OAC used. The limitations of the ORBIT score have been recently discussed^21^.

Despite this aim, one major limitation of the ORBIT and other bleeding risk scores (apart from HAS-BLED) is the exclusion of labile anticoagulation control (as reflected by time in therapeutic range [TTR]) amongst vitamin K antagonist (VKA, *e.g.* warfarin) users, despite the very strong association of poor TTR with major bleeding^3^,^22^,^23^. The VKAs are still in very widespread clinical use as OAC therapy worldwide, and clinically useful bleeding risk scores also need to be applicable to VKA users.

Recently we investigated the impact of TTR when added to the ORBIT and ATRIA bleeding risk scores, when compared to HAS-BLED, in predicting ‘clinically relevant bleeding’ in the AMADEUS Trial cohort^24^. Adding TTR to both ORBIT and ATRIA bleeding scores improved their predictive ability, although this analysis was hampered by a short follow-up observation, low number of adverse events and a broad definition of ‘*any clinically relevant bleeding’* rather than focusing on major bleeding^24^. Thus, a separate validation study in an *independent* cohort which is *adequately powered for major bleeding* is needed to confirm our initial observations.

The objectives for the present analysis were as follows: (i) to perform a comprehensive comparison of the four AF-validated bleeding risk scores (HAS-BLED, ORBIT, ATRIA and HEMORR_2_HAGES), amongst a large cohort of non-valvular AF patients; and (ii) to further investigate if the predictive value of bleeding risk scores other than HAS-BLED could be improved by incorporating TTR, if VKA was used.

## Methods

We tested the HAS-BLED, ORBIT, ATRIA and HEMORR_2_HAGES bleeding scores on the patients receiving warfarin in the pooled population dataset from the Stroke Prevention using an Oral Thrombin Inhibitor in patients with atrial Fibrillation (SPORTIF) III and V studies^25^,^26^,^27^. The SPORTIF trials were two multicentre phase III clinical trials comparing the efficacy and safety of the direct thrombin inhibitor ximelagatran, compared to warfarin, in predicting thromboembolic stroke in non-valvular AF patients.

De-identified datasets with patient-level information for the SPORTIF trials were obtained directly from Astra Zeneca, and all the analyses were performed independent of the company. All patients assigned to the warfarin treatment arms and with available data for the clinical variables used to calculate the four bleeding prediction scores were included in the present analysis. Detailed methods about evaluation of anticoagulation control, assessment of the HAS-BLED, ORBIT, ATRIA and HEMORR_2_HAGES bleeding scores and study outcomes definition are reported in the web-only Supplementary Material.

We considered ‘major bleeding’ events in two distinct ways, as follows: (i) “investigator level” events (that included the crude number of all the major bleeding events reported by any investigator at every study site); and (ii) “adjudicated events” (corresponding to the final trial adjudicated major bleeding events, after the independent central adjudication committee evaluated all the reported events). This distinction was done in order to analyse the ability of bleeding scores in correctly identifying patients at low risk of bleeding, when accounting for possible bleeding events that could occur in “real-life” management of AF patients (ie. at the ‘prescriber’ or investigator level), in contrast to the strictly defined adjudicated trial protocol criteria.

### Statistical Analysis

All continuous variables were tested for normality with the Shapiro-Wilk test. Variables with normal distribution were expressed as means and standard deviation, and tested for differences using a t-test. Non-parametric variables were expressed as median and interquartile range (IQR), with differences tested using the Mann-Whitney U test. Categorical variables, expressed as counts and percentages, were analysed by chi-squared test. A logistic regression analysis was performed to investigate the association between the “low risk” bleeding category and the clinical endpoint of “absence of major bleeding”. This analysis was performed according to the *“investigator level”* major bleeding events group.

Event-free survival analysis, assessed by an intention-to-treat approach, was performed according to bleeding risk categories and differences in survival distributions between subgroups were analysed using the log-rank test. A Cox proportional hazards analysis was used to evaluate the occurrence of major bleeding according to bleeding risk categories, based on *“adjudicated events”.*

A receiver operating characteristic (ROC) curve was compiled for all risk scores, using the major bleeding “adjudicated events” group, in order to evaluate the predictive ability of all models. Comparisons of ROC curves were performed according to De Long, De Long and Clarke-Pearson method^28^. Continuous net reclassification improvement (NRI) and integrated discriminatory improvement (IDI) were computed using the “PredictABEL” R package, according to methods described by Pencina *et al.*^29^.

To evaluate the impact of poor anticoagulation control amongst the bleeding scores, an additional analysis adding one point for TTR < 65%, was added to the ORBIT, ATRIA and HEMORR_2_HAGES scores to determine if this attributed any improvement in predictive performance for major bleeding.

Two-sided p values < 0.05 were considered statistically significant. All analyses were performed using SPSS v. 22.0 (IBM, NY, USA), MedCalc v. 15.6 (MedCalc Software, Belgium) and R for Mac OS X v. 3.2.1 (The R Foundation for Statistical Computing).

## Results

In the original combined SPORTIF dataset, a total of 3,665 patients were assigned to the warfarin treatment arm; data to calculate the bleeding risk scores for the present analyses were available in 3,551 patients (96.9%). The majority of patients were male (69.5%) and the median [IQR] age was 72 [66–77] years. A total of 706 (19.9%) patients were treated concomitantly with aspirin, while only 20.1% of patients were VKA naïve at baseline. Median [IQR] CHA_2_DS_2_-VASc score was 3 [2–4], with 3,074 (86.6%) patients being categorised as ‘high risk’.

### Bleeding Risk in Overall Population

**Figure 1:**
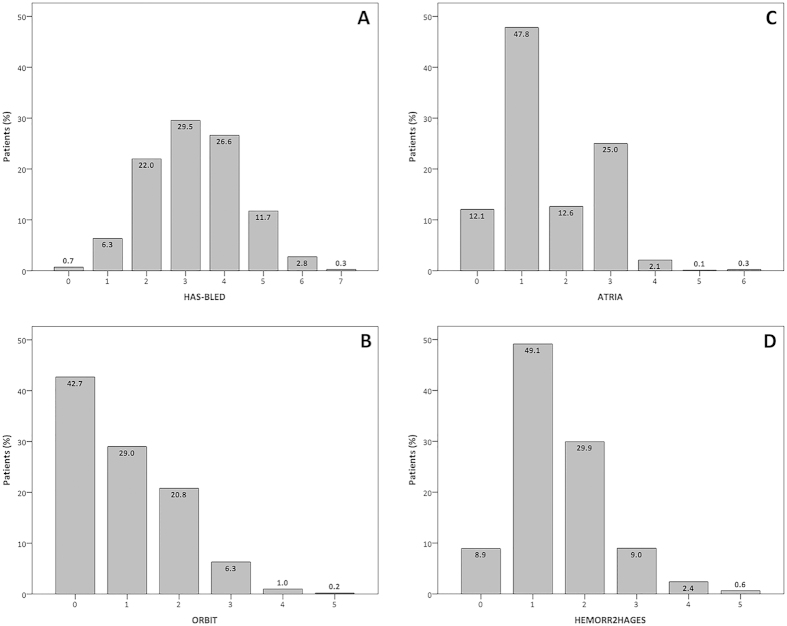
Distribution of scores for the cohort utilising each bleeding risk score.

Median [IQR] value in the overall study cohort for HAS-BLED score was 3 [2–4], while the median ORBIT, ATRIA and HEMORR_2_HAGES scores were 1 [0–2], 1 [1–3] and 1 [1, 2], respectively. Distribution of patients according to the various scores on each bleeding risk schema is shown in Fig. 1 (Panels A–D). High bleeding risk according to HAS-BLED (score ≥ 3) was seen in 71.0% of patients; whilst 7.5% of patients were at medium/high bleeding risk using the ORBIT score. The proportion of medium/high risk patients were 2.5% for ATRIA and 41.9% for HEMORR_2_HAGES.

### Clinical Characteristics at Baseline

Distribution of clinical characteristics according to risk categories for the various bleeding schemes are reported in eTable 1. Higher proportions of females were found in high or medium/high risk categories (p = 0.001 for HAS-BLED and p < 0.001 for the other scores). Coronary heart disease was more frequent in the high or medium/high risk categories for HAS-BLED (p < 0.001), ORBIT (p < 0.001) and HEMORR_2_HAGES (p = 0.026) but not for ATRIA (p = 0.13). No significant difference was found in previous stroke/transient ischemic attack between low and medium/high ATRIA categories (p = 0.065). Similarly, no difference in heart failure was found between the ORBIT and ATRIA score categories (p = 0.091 and p = 0.477, respectively), or in hypertension for ORBIT score categories (p = 0.874). Thromboembolic risk progressively increased between risk categories for all the scores.

The proportion of patients with good anticoagulation control (TTR > 70%) progressively decreased with increasing risk categories for all scores, except for ATRIA (p = 0.424). The HAS-BLED low risk category had the highest proportion of good anticoagulation control patients (64.7% vs 38.7% in high risk category, p < 0.001).

### Follow-Up Analysis

A median [IQR] follow-up of 1.6 [1.3–1.8] years yielded a total of 5,002 patient-years observation. A total of 162 “investigator level” major bleeding events were recorded. Of these, 127 were validated as “adjudicated events”, with an overall incidence of 2.5 per 100 patient-years. Event rates according to the various bleeding score and risk categories are shown in Table 1.

Major bleeding rates, both in the “investigator level” and “adjudicated event” groups, progressively *increased* as the bleeding risk score increased (and by bleeding risk categories) using the HAS-BLED score. Conversely, event rates *decreased* as the bleeding risk score increased and from low to medium/high bleeding risk categories, both for the ORBIT and ATRIA scores.

Based on the HEMORR_2_HAGES score, most of the events occurred in the ‘low risk’ category. Of the 127 *adjudicated* major bleeding events, 21.3% of events occurred in the ‘low risk’ HAS-BLED category (1.8 per 100 patient-years), compared to 87.4% occurring in the low risk category for ORBIT, 96.6% for ATRIA and 52.0% for HEMORR_2_HAGES (approx. 2.5 per 100 patient-years) (Table 1).

### Regression and Survival Analyses

Logistic regression analysis for the *absence* of any ‘investigator level’ defined major bleeding events, found that the HAS-BLED ‘low risk’ category was associated with the absence of any ‘investigator level’ defined major bleeding events (low risk vs. high risk, adjusted odds ratio [OR]: 1.46, 95% confidence interval [CI]: 1.00–2.13, p = 0.050). None of the other scores showed a significant association with the *absence* of any ‘investigator level’ defined major bleeding.

Analysis of bleeding scores as continuous variables, adjusted for sex and AF type, showed a significant association with *adjudicated* major bleeding events for all scores (eTable 2). Survival analysis showed that patients in high or medium/high risk category had a higher risk for adjudicated major bleeding events for both the HAS-BLED (Log-Rank: 5.147, p = 0.023) and ORBIT (Log-Rank: 5.247, p = 0.022) scores. Log-rank analyses showed no significant differences between risk categories for both ATRIA and HEMORR_2_HAGES scores.

**Figure 2:**
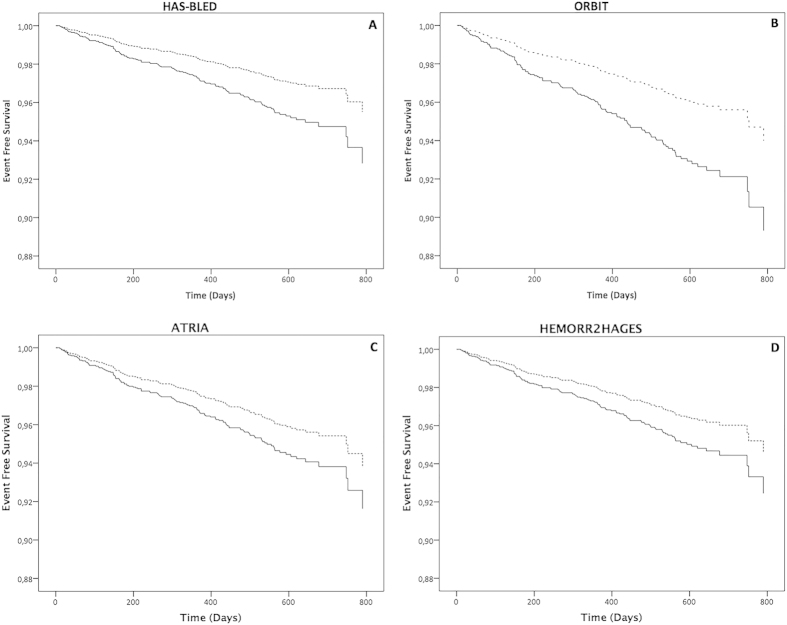
Event free survival for “adjudicated event” major bleeding according to risk categories for each bleeding risk score. Panel (**A**) HAS-BLED Solid Line = High Risk; Dashed Line = Low Risk; Panel (**B**) ORBIT Solid Line = Medium/High Risk; Dashed Line = Low Risk; Panel (**C**) ATRIA Solid Line = Medium/High Risk; Dashed Line = Low Risk; Panel (**D**) HEMORR_2_HAGES Solid Line = Medium/High Risk; Dashed Line = Low Risk.

After adjustment for gender and type of AF, Cox regression analyses demonstrated that a high or medium/high risk category was significantly associated with adjudicated major bleeding for both HAS-BLED (HR: 1.62, 95% CI: 1.06–2.48, p = 0.026) [Fig. 2, Panel A] and ORBIT (HR: 1.83, 95% CI: 1.08–3.09, p = 0.024) [Fig. 2, Panel B], but not for the ATRIA (HR: 1.36, 95% CI: 0.50–3.69, p = 0.544) [Fig. 2, Panel C] and HEMORR_2_HAGES (HR: 1.41, 95% CI: 0.99–2.00, p = 0.057) [Fig. 2, Panel D] scores.

### Performance and Reclassification Analysis

Receiver operating characteristic (ROC) curves analysis (eTable 3) showed that all the four scores were able to identify AF patients that reported an adjudicated major bleeding event. ROC curves comparison analyses showed that HEMORR_2_HAGES had a worse performance compared both to ORBIT (z: 1.923, p = 0.054) and ATRIA (z: 2.521, p = 0.012) scores. No other statistically significant differences were found between the other risk scores. Based on Pencina *et al.*^29^, there was a significant negative reclassification with the HEMORR_2_HAGES score compared with ORBIT and ATRIA (NRI: −0.2164, p = 0.016 and NRI: −0.3128, p < 0.001 respectively) and a loss in sensitivity in comparison with all the other bleeding scores based on IDI analyses (Table 2).

### Impact of TTR

Modified scores, as continuous variables, were significantly associated (p < 0.001) with adjudicated major bleeding events (eTable 4). When considered as categorical variables, medium/high risk vs. low risk was also significantly associated with adjudicated events (p < 0.001 for ORBIT and ATRIA; p = 0.013 for HEMORR_2_HAGES).

Comparisons between ROC curves for the modified scores compared with their original values are summarised in Table 3, and show a significant difference for the HEMORR_2_HAGES (p = 0.028) score with borderline significance for the modified ATRIA score (p = 0.052). Reclassification analysis demonstrated that by adding TTR < 65%, all 3 modified scores reported a significant improvement in reclassification and discrimination gain, with significant differences in NRI and IDI compared to their original scores that did not include TTR (Table 3). No significant difference was found in AUC for each of the modified scores (that included TTR), when compared with HAS-BLED (full data not shown).

## Discussion

Our principal finding was that the different bleeding scores provided different discriminatory capacities for major bleeding in anticoagulated AF patients; specifically, both HAS-BLED and ORBIT categorised adjudicated major bleeding events in low risk and high or medium/high risk patients appropriately, but the majority of adjudicated major bleeding events occurred in the ‘low risk’ ORBIT category. Second, adding a labile INR criterion (TTR < 65%) to ORBIT, ATRIA and HEMORR_2_HAGES led to improved predictive performance for major bleeding compared to the original scores. Thus, both the ATRIA and ORBIT scores may perform suboptimally in identifying serious bleeding risk in a patient on warfarin, unless they are re-calibrated taking labile INRs (or TTRs) into consideration.

In contrast, the HAS-BLED score already considers ‘labile INR’ as one of its criteria, which is applicable only for a VKA user (whilst the L criterion is not applicable if a NOAC is used). The HAS-BLED score has been well-validated in predicting major bleeding in various clinical settings^11^,^12^,^13^,^30^. This score has been tested in untreated, aspirin, VKA^11^ and in non-VKA anticoagulant settings^16^,^17^,^31^, as well as in AF and non-AF cohorts. HAS-BLED is also predictive of major bleeding during bridging^13^ and in the setting of acute coronary syndrome and percutaneous coronary intervention^30^. Previous direct comparisons with HEMORR_2_HAGES^15^ and ATRIA^14^ have showed that HAS-BLED was a good as (or even superior) in the evaluation of bleeding risk^11^,^17^,^32^, even in ‘real world’ settings^33^ and in predicting intracranial haemorrhage (ICH)^34^.

The ORBIT score was derived from a large industry-sponsored observation registry that enrolled more than 7,400 AF patients^19^. The authors proposed that the development of this new bleeding score would allow the evaluation of bleeding risk in AF patients in an easier and simplified manner compared with other scores, taking into consideration clinical variables easily collectible from clinical history. The score was validated in the ROCKET-AF trial, which was a trial of anticoagulation with rivaroxaban vs. warfarin, among high risk AF patients (only those with CHADS_2_ score ≥2 were included, and those with score = 2 were capped at 10%)^35^. Based on the published results the ORBIT score performed similarly in the derivation cohort and statistically better than other validated scores, HAS-BLED and ATRIA^20^. As highlighted in the accompanying Editorial^21^, statistical significance and clinical applicability need to be balanced^36^. For example, a 40 year old man with prior stroke, labile INRs on warfarin (*e.g.* TTR 50%), concomitant use of non-steroidal anti-inflammatory drugs, abnormal liver function would have an ORBIT score of 0 (*i.e.* low risk), but would have a HAS-BLED score of 4 (high risk)^21^. As recommended in guidelines, the responsible physician would ‘flag up’ this patient with high HAS-BLED score, and in accordance with good clinical practice would strive to control blood pressure, optimise the TTR (or swap to a NOAC), and reduce concomitant drugs assumption. The ORBIT score would not ‘flag up’ such a patient (relevant to automated ‘alert flags’ used in electronic health records) nor draw attention to the reversible bleeding risk factors.

Our study seems to confirm the illustrative case above, given that use of HAS-BLED categorised adjudicated major bleeding events in low risk and high risk patients appropriately, whilst the majority of major bleeding events occurred in patients categorised as ‘low risk’ using the ORBIT score. Also, HAS-BLED score category was associated with the *absence* of any “investigator defined” major bleeding events, whilst risk categorisation using the ORBIT score was not significantly associated with the *absence* of any investigator defined major bleeding events.

Consideration of poor anticoagulation control is crucial when evaluating bleeding risk. Our data clearly show an improved association with adjudicated major bleeding events and risk stratification for the ORBIT, ATRIA and HEMORR_2_HAGES scores when adding TTR < 65% as a measure of poor anticoagulation, with improved reclassification and discriminatory performance. Moreover, these results clearly confirm and further strengthen our previous analyses. In the AMADEUS trial cohort, adding TTR to the ORBIT and ATRIA bleeding risk score schemes improved their predictive abilities for clinically relevant bleeding^24^. Despite that, given that the AMADEUS trial was stopped early, and given the few major bleeding endpoints during the short follow-up period, the analysis was only focused on ‘any clinically relevant bleeding’.

Our study reinforces the concept that neglecting anticoagulation control, expressed by TTR, in bleeding assessment would led to a reduced performance of bleeding prediction scores in patients treated with VKA. This paper has important clinical implications as it suggests that some bleeding scores (ORBIT and ATRIA) are suboptimally in identifying serious bleeding risk in a patient on VKA, unless they are re-calibrated taking labile INRs (or TTRs) into consideration. Indeed, VKAs are still very commonly used worldwide as oral anticoagulants and despite the introduction of the NOACs, over-simplification of bleeding risk scores advocated to work for both VKAs and NOACs, but yet ignoring TTR in the VKA users, could potentially underestimate bleeding risks (and lead to potentially serious bleeding events). Of note, the HAS-BLED score already assigns 1 point for ‘labile INR’ and there was no difference in AUCs for the modified scores (adding TTR to HEMORR_2_HAGES, ORBIT and ATRIA), when compared with HAS-BLED.

### Limitations

This study is mainly limited by the post-hoc, retrospective nature of our analysis and by the relatively short follow-up observation period, eventhough our study population was an ancillary analysis to a well conducted prospective randomised trial with adjudicated endpoints. Moreover, not all the factors (e.g. genetic factors for HEMORR_2_HAGES) required for scores assessment were present in our data set. In addition, we used only the Cockroft-Gault creatinine clearance calculation for renal function assessment, differently from the definitions used in the original score schemes. Also, study patients were carefully followed up as per the clinical trial protocol, risk factors would have been proactively managed, and some patients at very high bleeding risk were excluded due to the trial exclusion criteria. These reasons could account for the low bleeding rates seen, and the low event rates even amongst ‘high risk’ patients. Nonetheless, the HAS-BLED score was developed to ‘flag up’ the patients *potentially* at high risk for bleeding for more careful review and follow-up so that reversible risk factors can be addressed (rather than let bleeding events actually occur).

## Conclusion

In conclusion, the HAS-BLED score had the best predictive value in identifying those at ‘low risk’ of major amongst bleeding VKA-treated patients. Indeed, the majority of such bleeds occurred in patients categorised as ‘low risk’ using the ORBIT, ATRIA and HEMORR_2_HAGES scores. Second, adding labile INR (*i.e.* TTR < 65%) to the ORBIT, ATRIA and HEMORR_2_HAGES bleeding risk scores led to their improved predictive performance for major bleeding.

## Additional Information

**How to cite this article**: Proietti, M. *et al.* Major Bleeding in Patients with Non-Valvular Atrial Fibrillation: Impact of Time in Therapeutic Range on Contemporary Bleeding Risk Scores. *Sci. Rep.* **6**, 24376; doi: 10.1038/srep24376 (2016).

## Supplementary Material

Supplementary Information

## Figures and Tables

**Table 1:** Major bleeding event rates according to the bleeding risk scores.

	Major Bleeding *Investigator Level* N = 162	Major Bleeding *Adjudicated Event*N = 127		Major Bleeding *Investigator Level*N = 162	Major Bleeding *Adjudicated Event*N = 127
HAS-BLED *N (*%)			ATRIA *N (*%)		
0	1 (0.6)	1 (0.8)	0	11 (6.8)	8 (6.3)
1	7 (4.3)	3 (2.4)	1	64 (39.5)	8 (37.8)
2	28 (17.3)	23 (18.1)	2	29 (17.9)	22 (17.3)
3	48 (29.6)	31 (24.4)	3	54 (33.3)	45 (35.4)
4	43 (26.5)	41 (32.3)	4	3 (1.9)	3 (2.4
5	26 (16.0)	20 (15.7)	5	0 (0.0)	0 (0.0)
6	9 (5.6)	8 (6.3)	6	1 (0.6)	1 (0.8)
HAS-BLED Risk *N (*%)			ATRIA Risk *N (*%)		
Low	36 (22.2)	27 (21.3)	Low	158 (97.5)	123 (96.9)
High	126 (77.8)	100 (78.7)	Medium/High	4 (2.5)	4 (3.1
HAS-BLED Risk (*per 100 patient-years*)			ATRIA Risk (*per 100 patient-years*)		
Low	2.4	1.8	Low	3.3	2.5
High	3.6	2.9	Medium/High	3.4	3.4
ORBIT *N* (%)			HEMORR_2_HAGES *N* (%)		
0	48 (29.6)	38 (29.9)	0	9 (5.6)	6 (4.7)
1	49 (30.2)	36 (28.3)	1	81 (50.0)	60 (47.2)
2	47 (29.0)	37 (29.1)	2	45 (27.8)	39 (30.7)
3	15 (9.3)	14 (11.0)	3	22 (13.6)	17 (13.4)
4	2 (1.2)	2 (1.6)	4	4 (2.5)	4 (3.1)
5	1 (0.6)	0 (0)	5	1 (0.6)	1 (0.8)
ORBIT Risk *N* (%)			HEMORR_2_HAGES Risk *N* (%)		
Low	144 (88.9)	111 (87.4)	Low	90 (55.6)	66 (52.0)
Medium/High	18 (11.1)	16 (12.6)	Medium/High	72 (44.4)	48 (48.0)
ORBIT Risk (*per 100 patient-years*)			HEMORR_2_HAGES Risk (*per 100 patient-years*)		
Low	3.1	2.4	Low	3.0	2.2
Medium/High	4.9	4.4	Medium/High	3.6	2.4

**Table 2:** Reclassification analysis for the various bleeding risk scores.

	*vs*HAS-BLED				*vs*ORBIT				*vs*ATRIA			
	*NRI*	*p*	*IDI*	*p*	*NRI*	*p*	*IDI*	*p*	*NRI*	*p*	*IDI*	*p*
HAS-BLED												
ORBIT	−0.0077	0.392	0	0.646								
ATRIA	−0.0883	0.323	0	0.611	0.0355	0.683	0	0.924				
HEMORR_2_HAGES	−0.1366	0.119	**−0.0018**	**0.039**	**−0.2164**	**0.016**	**−0.0023**	**0.006**	**−0.3128**	**<0.001**	**−0.0024**	**0.003**

IDI = integrated discriminatory improvement; NRI = net reclassification improvement.

**Table 3:** Comparison of ROC curves and reclassification analysis for modified bleeding scores with TTR.

	AUC	p*	NRI	p	IDI	p
	*vs* ORBIT					
ORBIT + TTR< 65%	0.609	0.106	0.2508	0.0054	0.0023	0.0092
	*vs* ATRIA					
ATRIA + TTR< 65%	0.611	0.052	0.250	0.0054	0.0020	0.0014
	*vs* HEMORR_2_HAGES					
HEMORR_2_HAGES + TTR< 65%	0.578	0.028	0.263	0.0034	0.0015	0.0016

AUC = area under the curve; IDI = integrated discriminatory improvement; NRI = net reclassification improvement. *z test for AUC comparison.
