# Supplementary material for: Major Bleeding in Patients with Non-Valvular Atrial Fibrillation: Impact of Time in Therapeutic Range on Contemporary Bleeding Risk Scores
Source: Sci Rep. 2016 Apr 12;6:24376. doi: 10.1038/srep24376 (PMC4828703; doi:10.1038/srep24376)
Supplement: Supplementary Information [file srep24376-s1.doc]

**Major Bleeding in Patients with Non-Valvular Atrial Fibrillation: Impact of Time in Therapeutic Range on Contemporary Bleeding Risk Scores**

Marco Proietti, Keitaro Senoo, Deirdre A Lane, Gregory YH Lip

*Detailed Methods and Supplementary Data*

**Detailed Methods** page. 2

**eTable 2**

Association between bleeding risk scores as continuous variable and adjudicated major bleeding events page. 5

**eTable 3**

AUC values for the various bleeding prediction score page. 6

**eTable 5**

Association of modified bleeding scores with adjudicated

major bleeding events page. 7

**Additional References**  page. 8

**Detailed Methods**

*Thromboembolic Risk and Anticoagulation Control*

Thromboembolic risk was categorised according to CHA_2_DS_2_-VASc score^1^. “Low risk” patients were defined as a CHA_2_DS_2_-VASc score=0 in males or 1 in females; “moderate risk” was defined as male patients with CHA_2_DS_2_-VASc=1; and “high risk” as a CHA_2_DS_2_-VASc score ≥2^2^.

Anticoagulation control, as reflected by TTR was calculated using the standardized Rosendaal interpolation method^3^. Good anticoagulation control was defined as a mean TTR>70%^4^. Compared to our previous paper^5^, for the purposes of the present analysis we used a more strict cut-off for the ‘labile international normalized ratio (INR)’ criterion. Thus, labile INR was defined for a mean TTR <65%, consistent with previous published evidence^6,7^.

*Assessment of Bleeding Risk Scores*

The HAS-BLED score was developed in 2010 to devise a simple and clinically-driven risk score to assess major bleeding risk in AF patients treated with VKA^8^. One point each was allocated for the presence of hypertension, impaired renal or liver function, history of stroke, history of bleeding, labile international normalized ration (INR), elderly (age>65 years), concomitant use of antiplatelet agents or non-steroidal anti-inflammatory drugs and alcohol consumption (more than 8 units a week). The “impaired renal function” variable was considered as a creatinine clearance <60 ml/min according to the Cockroft-Gault formula. The “impaired liver function” criterion was scored 0, since liver dysfunction was an exclusion criterion from the original trial protocol. According to European Society of Cardiology a HAS-BLED score ranging from 0 to 2 was categorized as “low risk”, while HAS-BLED score ≥3 was categorised as “high risk”^9^.

The ORBIT score was developed from the “Outcomes registry for better informed treatment of atrial fibrillation” study cohort^10,11^, and calculated as follows: 1 point each for Age older than 74, insufficient kidney function (defined as estimated glomerular filtration rate below 60 mg/dL/1.73 m^2^) and treatment with any antiplatelet drug, while 2 points were assigned to a positive clinical history for bleeding and the presence of anaemia or abnormal haemoglobin (<13 mg/dL for males and <12 mg/dL for females) or reduced haematocrit (<40% for males and <36% for females). For this analysis, 0 was assigned for the “anaemia” variable, as this was an exclusion criterion in the original SPORTIF trial protocol. “Insufficient kidney function” criterion was defined as reported above for the HAS-BLED score. An ORBIT score ≤2 was categorised as “low risk”, while an ORBIT score ≥3 was categorised as “medium/high risk”.

The ATRIA score was developed in 2011 from a derivation cohort of the “Anticoagulation and Risk Factors in Atrial Fibrillation” study, a large nationwide observational cohort on AF patients among the general population^12^. According to the original design, three points were assigned for the presence of anaemia or concomitant diagnosis of a severe renal disease (estimated glomerular filtration rate<30 ml/min), 2 points for age ≥75 years and one point for a positive clinical history of bleeding or diagnosis of hypertension. As reported above, 0 was assigned for the “anaemia” criterion as anaemic patients were excluded from participation in the SPORTIF trials. A total score <4 points was categorized as “low risk”, while a score ≥4 was defined as “medium/high risk”.

The HEMORR_2_HAGES score was developed in 2006 and derived from the “National Registry of Atrial Fibrillation”, comprised of AF patients within a Medicare programme^13^. The original model allocated one point for the following: hepatic or renal disease, ethanol abuse, malignancy, older age (≥75 years), reduced platelet count or function, hypertension, anaemia, genetic factors, excessive fall risk, history of stroke. Two points were assigned for a positive bleeding history. For the present analyses we considered “alcohol abuse” as the consumption of >20 units of alcohol per week; due to the absence of data about genetic factors, this item was not considered for the score calculation. Presence of hypertension, older age, previous bleeding and history of stroke were awarded 1 point for each, while for all of the other criteria 0 points was assigned, since these factors were considered as exclusion criteria in the original SPORTIF study protocol. Patients were categorized as low risk scores of 0-1 points, while medium/high bleeding risk was defined as a score of ≥2.

*Study Outcomes*

The study endpoint was the occurrence of major bleeding defined by ≥1 of the following criteria: clinically overt bleeding with a concomitant fall in haemoglobin levels more than 2 g/dL or requiring blood transfusion of at least 2 units of whole blood or erythrocytes; bleeding episode involving a critical site (intracranial, intraspinal, intraocular, retroperitoneal, pericardial or non-traumatic intra-articular bleeding) ^14^.

**eTable 1**: Demographic and clinical characteristics according to bleeding risk score categories

|  | **HAS-BLED Risk** | | ***p*** | **ORBIT Risk** | | ***p*** |
| --- | --- | --- | --- | --- | --- | --- |
|  | ***Low*** | ***High*** |  | ***Low*** | ***Medium/High*** |  |
| **N (%)** | *N=1,031* | *N=2,520* |  | *N=3,285* | *N=266* |  |
| **Age** *years*  Median [IQR] | 65 [60-74] | 73 [68-78] | <0.001 | 71 [65-76] | 79 [76-82] | <0.001 |
| **Gender**  *Male*  *Female* | 756 (73.3)  275 (26.7) | 1,711 (67.9)  809 (32.1) | 0.001 | 2,314 (70.4)  971 (29.6) | 153 (57.5)  113 (42.5) | <0.001 |
| **Current Smoker** | 110 (10.7) | 212 (8.4) | 0.034 | 311 (9.5) | 11 (4.1) | 0.004 |
| **Alcohol Habit** | 278 (27.0) | 1,326 (52.6) | <0.001 | 1,504 (45.8) | 100 (37.6) | 0.010 |
| **Chronic Atrial Fibrillation*** | 934 (90.6) | 2,234 (88.7) | 0.102 | 2,930 (89.2) | 238 (89.5) | 0.909 |
| **BMI** *kg/cm^2^*†  Median [IQR] | 29.4 [26.0-33.6] | 27.5 [24.7-31.1] | <0.001 | 28.3 [25.2-32.0] | 25.7 [23.7-27.9] | <0.001 |
| **Hypertension** | 608 (59.0) | 2,116 (84.0) | <0.001 | 2,521 (76.7) | 203 (76.3) | 0.874 |
| **Diabetes Mellitus** | 244 (23.7) | 589 (23.4) | 0.851 | 770 (23.4) | 63 (23.7) | 0.913 |
| **Creatinine Clearance** *ml/min*  Median [IQR] | 93.0 [75.0-118.6] | 71.3 [54.3-93.9] | <0.001 | 80.8 [62.2-104] | 53.0 [45.3-62.0] | <0.001 |
| **Coronary Heart Disease** | 391 (37.9) | 1,184 (47.0) | <0.001 | 1,429 (43.5) | 146 (54.9) | <0.001 |
| **Stroke/TIA** | 116 (11.3) | 614 (24.4) | <0.001 | 649 (19.8) | 81 (30.5) | <0.001 |
| **Chronic Heart Failure** | 448 (43.5) | 876 (34.8) | <0.001 | 1,212 (36.9) | 112 (42.1) | 0.091 |
| **Clinically Relevant Bleeding** | 11 (1.1) | 189 (7.5) | <0.001 | 74 (2.3) | 126 (47.4) | <0.001 |
| **Prior Aspirin Use** | 73 (7.1) | 633 (25.1) | <0.001 | 534 (16.3) | 172 (64.7) | <0.001 |
| **Prior Vitamin K-Antagonist Use** | 893 (86.6) | 1,916 (76.0) | <0.001 | 2,650 (80.7) | 159 (59.8) | <0.001 |
| **TTR>70%** | 667 (64.7) | 975 (38.7) | <0.001 | 1,540 (46.9) | 102 (38.3) | 0.025 |
| **CHA_2_DS_2_-VASc**  Median [IQR] | 2 [1-3] | 3 [2-4] | <0.001 | 3 [2-4] | 4 [3-5] | <0.001 |
| **Thromboembolic Risk**  *Low*  *Intermediate*  *High* | 6 (0.6)  278 (27.0)  747 (72.5) | 0 (0.0)  193 (7.7)  2,327 (92.3) | <0.001 | 6 (0.2)  468 (14.2)  2,811 (85.6) | 0 (0.0)  3 (1.1)  263 (98.9) | <0.001 |

**Legend:** IQR= interquartile range; TIA= transient ischemic attack; TTR= time in therapeutic range; *3549 patients; †3541 patients.

**eTable 1 (continued)**: Demographic and clinical characteristics according to bleeding risk score categories

|  | **ATRIA Risk** | | ***p*** | **HEMORR_2_HAGES Risk** | | ***p*** |
| --- | --- | --- | --- | --- | --- | --- |
|  | ***Low*** | ***Medium/High*** |  | ***Low*** | ***Medium/High*** |  |
| **N (%)** | *N=3,463* | *N=88* |  | *N=2,062* | *N=1,489* |  |
| **Age** *years*  Median [IQR] | 72 [65-77] | 79 [76-82] | <0.001 | 68 [63-73] | 77 [73-80] | <0.001 |
| **Gender**  *Male*  *Female* | 2,421 (69.9)  1,042 (30.1) | 46 (53.2)  42 (47.7) | <0.001 | 1,537 (74.5)  525 (25.5) | 930 (62.5)  559 (37.5) | <0.001 |
| **Current Smoker** | 317 (9.2) | 5 (5.7) | 0.263 | 225 (10.9) | 97 (6.5) | <0.001 |
| **Alcohol Habit** | 1,568 (45.3) | 36 (40.9) | 0.416 | 967 (46.9) | 637 (42.8) | 0.015 |
| **Chronic Atrial Fibrillation*** | 3,088 (89.2) | 80 (90.9) | 0.614 | 1,836 (89.0) | 1,332 (89.6) | 0.610 |
| **BMI** *kg/cm^2^*†  Median [IQR] | 28.1 [25.1-31.7] | 26.9 [23.3-29.0] | <0.001 | 28.7 [25.6-32.6] | 27.3 [24.4-30.7] | <0.001 |
| **Hypertension** | 2,640 (76.2) | 84 (95.5) | <0.001 | 1,357 (65.8) | 1,367 (91.8) | <0.001 |
| **Diabetes Mellitus** | 812 (23.4) | 21 (23.9) | 0.928 | 474 (23.0) | 359 (24.1) | 0.436 |
| **Creatinine Clearance** *ml/min*  Median [IQR] | 79.3 [59.8-103.0] | 60.4 [44.2-74.7] | <0.001 | 87.7 [69.2-112.7] | 65.1 [51.7-84.6] | <0.001 |
| **Coronary Heart Disease** | 1,529 (44.2) | 46 (52.3) | 0.130 | 882 (42.8) | 693 (46.5) | 0.026 |
| **Stroke/TIA** | 705 (20.4) | 25 (28.4) | 0.065 | 94 (4.6) | 636 (42.7) | <0.001 |
| **Chronic Heart Failure** | 1,288 (37.2) | 36 (40.9) | 0.477 | 811 (39.3) | 513 (34.5) | 0.003 |
| **Clinically Relevant Bleeding** | 127 (3.7) | 73 (83.0) | <0.001 | 0 (0.0) | 200 (13.4) | <0.001 |
| **Prior Aspirin Use** | 696 (20.1) | 10 (11.4) | 0.043 | 378 (18.3) | 328 (22.0) | 0.006 |
| **Prior Vitamin K-Antagonist Use** | 2,741 (79.2) | 68 (77.3) | 0.669 | 1,627 (78.9) | 1,182 (79.4) | 0.729 |
| **TTR>70%** | 1,605 (46.3) | 37 (42.0) | 0.424 | 982 (47.6) | 660 (44.3) | 0.052 |
| **CHA_2_DS_2_-VASc**  Median [IQR] | 3 [2-4] | 4 [3-5] | <0.001 | 2 [2-3] | 4 [3-5] | <0.001 |
| **Thromboembolic Risk**  *Low*  *Intermediate*  *High* | 6 (0.2)  471 (13.6)  2,986 (86.2) | 0 (0.0)  0 (0.0)  88 (100.0) | 0.001 | 6 (0.3)  440 (21.3)  1,616 (78.4) | 0 (0.0)  31 (2.1)  1,458 (97.9) | <0.001 |

**Legend:** IQR= interquartile range; TIA= transient ischemic attack; TTR= time in therapeutic range; *3549 patients; †3541 patients.

**eTable 2:** Association between bleeding risk scores as continuous variable and adjudicated major bleeding events

|  | **HR*** | **95% CI** | **p** |
| --- | --- | --- | --- |
| **HAS-BLED** | 1.30 | 1.13-1.50 | <0.001 |
| **ORBIT** | 1.36 | 1.17-1.59 | <0.001 |
| **ATRIA** | 1.35 | 1.16-1.57 | <0.001 |
| **HEMORR_2_HAGES** | 1.24 | 1.04-1.48 | 0.018 |

**Legend:** CI= confidence interval; HR= hazard ratio. *as per every score point.

**eTable 3:** AUC values for the various bleeding prediction scores

|  | **AUC** | **95% CI** | **z statistic** | **p** |
| --- | --- | --- | --- | --- |
| **HAS-BLED** | 0.581 | 0.564-0.597 | 3.215 | 0.001 |
| **ORBIT** | 0.589 | 0.573-0.606 | 3.596 | <0.001 |
| **ATRIA** | 0.590 | 0.574-0.606 | 3.813 | <0.001 |
| **HEMORR_2_HAGES** | 0.549 | 0.532-0.565 | 2.036 | <0.001 |

**Legend:** AUC= area under the curve; CI= confidence interval.

**eTable 4:** Association of the modified bleeding scores (by addition of labile INR, defined as Time in Therapeutic Range <65%) with adjudicated major bleeding events

|  | **HR** | **95% CI** | **p** |
| --- | --- | --- | --- |
| **ORBIT+TTR<65%** *(as continuous variable)* | 1.43 | 1.24-1.64 | <0.001 |
| **ORBIT+TTR<65%** *(medium/high vs. low risk)* | 2.19 | 1.49-3.24 | <0.001 |
| **ATRIA+TTR<65%** *(as continuous variable)* | 1.47 | 1.27-1.69 | <0.001 |
| **ATRIA+TTR<65%** *(medium/high vs. low risk)* | 2.44 | 1.63-3-67 | <0.001 |
| **HEMORR_2_HAGES+TTR<65%** *(as continuous variable)* | 1.34 | 1.15-1.56 | <0.001 |
| **HEMORR_2_HAGES+TTR<65%** *(medium/high vs. low risk)* | 1.62 | 1.11-2.37 | 0.013 |

**Legend:** CI= confidence interval; HR= hazard ratio; TTR= time in therapeutic range.

**Additional References**

1. Lip, G. Y. H., Nieuwlaat, R., Pisters, R., Lane, D. A. & Crijns, H. J. G. M. Refining clinical risk stratification for predicting stroke and thromboembolism in atrial fibrillation using a novel risk factor-based approach: the euro heart survey on atrial fibrillation. *Chest* **137,** 263–72 (2010).

2. Lip, G. Y. H., Skjøth, F., Rasmussen, L. H. & Larsen, T. B. Oral Anticoagulation, Aspirin, or No Therapy in Patients With Nonvalvular AF With 0 or 1 Stroke Risk Factor Based on the CHA2DS2-VASc Score. *J. Am. Coll. Cardiol.* **65,** 1385–94 (2015).

3. Rosendaal, F. R., Cannegieter, S. C., van der Meer, F. J. & Briët, E. A method to determine the optimal intensity of oral anticoagulant therapy. *Thromb. Haemost.* **69,** 236–9 (1993).

4. De Caterina, R. *et al.* Vitamin K antagonists in heart disease: Current status and perspectives (Section III). *Thromb. Haemost.* **110,** 1087–1107 (2013).

5. Senoo, K., Proietti, M., Lane, D. A. & Lip, G. Y. H. Evaluation of the HAS-BLED, ATRIA and ORBIT bleeding risk scores in atrial fibrillation patients on warfarin. *Am. J. Med.* (2015). doi:10.1016/j.amjmed.2015.10.001

6. Connolly, S. J. *et al.* Benefit of oral anticoagulant over antiplatelet therapy in atrial fibrillation depends on the quality of international normalized ratio control achieved by centers and countries as measured by time in therapeutic range. *Circulation* **118,** 2029–37 (2008).

7. Gallego, P. *et al.* Cessation of oral anticoagulation in relation to mortality and the risk of thrombotic events in patients with atrial fibrillation. *Thromb. Haemost.* **110,** 1189–1198 (2013).

8. Pisters, R. *et al.* A novel user-friendly score (HAS-BLED) to assess 1-year risk of major bleeding in patients with atrial fibrillation: the Euro Heart Survey. *Chest* **138,** 1093–100 (2010).

9. Camm, A. J. *et al.* 2012 focused update of the ESC Guidelines for the management of atrial fibrillation: an update of the 2010 ESC Guidelines for the management of atrial fibrillation. Developed with the special contribution of the European Heart Rhythm Association. *Eur. Heart J.* **33,** 2719–47 (2012).

10. Piccini, J. P. *et al.* Outcomes registry for better informed treatment of atrial fibrillation: rationale and design of ORBIT-AF. *Am. Heart J.* **162,** 606–612.e1 (2011).

11. O’Brien, E. C. *et al.* The ORBIT bleeding score: a simple bedside score to assess bleeding risk in atrial fibrillation. *Eur. Heart J.* (2015). doi:10.1093/eurheartj/ehv476

12. Fang, M. C. *et al.* A new risk scheme to predict warfarin-associated hemorrhage: The ATRIA (Anticoagulation and Risk Factors in Atrial Fibrillation) Study. *J. Am. Coll. Cardiol.* **58,** 395–401 (2011).

13. Gage, B. F. *et al.* Clinical classification schemes for predicting hemorrhage: results from the National Registry of Atrial Fibrillation (NRAF). *Am. Heart J.* **151,** 713–9 (2006).

14. Halperin, J. L. Ximelagatran compared with warfarin for prevention of thromboembolism in patients with nonvalvular atrial fibrillation: Rationale, objectives, and design of a pair of clinical studies and baseline patient characteristics (SPORTIF III and V). *Am. Heart J.* **146,** 431–8 (2003).
